# Bioelectrical Impedance-Based Time-Domain Analysis for Cerebral Autoregulation Assessment

**DOI:** 10.3390/s25185762

**Published:** 2025-09-16

**Authors:** Yimin Zhou, Wei He, Bin Yang, Xuetao Shi, Yifan Liu, Yanyan Shi, Feng Fu

**Affiliations:** 1 Department of Biomedical Engineering, The Fourth Military Medical University, Xi’an 710032, China; zhouyimin_7776@163.com (Y.Z.);; 2 Shaanxi Key Laboratory for Bioelectromagnetic Detection and Intelligent Perception, Xi’an 710032, China; 3 Department of Radiation Protection Medicine, Faculty of Preventive Medicine, The Fourth Military Medical University, Xi’an 710032, China; afmmuhw@163.com; 4 Ministry of Education Key Lab of Hazard Assessment and Control in Special Operational Environment, Xi’an 710032, China; 5 College of Electronic and Electrical Engineering, Henan Normal University, Xinxiang 453007, China

**Keywords:** cerebral autoregulation, bioelectrical impedance, rheoencephalography, time-domain analysis, wearable sensor, non-invasive monitoring

## Abstract

**Highlights:**

**What are the main findings?**

- A novel, non-invasive method using bioelectrical impedance was developed to assess cerebral autoregulation in real time.
- The time constant (*τ*_REG_) successfully differentiated autoregulatory capacity between young and middle-aged healthy adults.

**What are the implications of the main findings?**

- *τ*_REG_ shows promise as a quantitative biomarker for age-related changes in cerebral autoregulation.
- This method may support early cerebrovascular risk detection and personalized monitoring strategies.

**Abstract:**

Cerebral autoregulation refers to the ability of cerebral vasculature to maintain stable blood flow by adjusting vascular resistance in response to changes in perfusion pressure. With advancing age, this regulatory capacity gradually declines, and its early, real-time, and dynamic monitoring holds potential as a promising approach for the prevention and treatment of cerebrovascular diseases. Given the absence of an established “gold standard” for assessing cerebral autoregulation, this study aimed to develop a non-invasive, continuous method for assessing cerebral autoregulation based on bioelectrical impedance technology. Using a wearable headband in combination with a Finapres device, blood pressure and cerebral blood flow were continuously monitored. A novel impedance recovery curve method was developed and, together with systemic blood pressure data, used to construct a hierarchical cerebral autoregulation assessment model via system identification. Moreover, the utility of this method in differentiating autoregulatory capacity across age groups (young adult and middle-aged) was assessed. The results demonstrated that the time constant (*τ*_REG_), which characterizes the speed of cerebral blood flow recovery, differed significantly between the young adult and middle-aged groups (*p* < 0.001). These findings suggest the potential of *τ*_REG_ as a quantitative indicator for distinguishing cerebral autoregulatory function between healthy age cohorts.

## 1. Introduction

Cerebral autoregulation refers to the ability of cerebral blood vessels to adjust their vascular resistance in response to changes in cerebral perfusion pressure, thereby maintaining constant cerebral blood flow [1]. Stable and sufficient cerebral blood flow is essential for sustaining normal brain function and meeting its high metabolic demands. Notably, cerebral autoregulation impairment has been identified as a significant prognostic predictor in patients with various acute neurological disorders [2,3,4]. This highlights the importance of investigating the pathogenesis of cerebrovascular diseases, identifying therapeutic targets, and enabling early warning through real-time dynamic monitoring to improve treatment efficacy. Abnormal cerebral hemodynamics are closely associated with the onset and progression of cerebrovascular diseases, highlighting the assessment of cerebral autoregulation as a potential promising approach for prevention and treatment. Current techniques and methodologies for evaluating cerebral autoregulation are diverse but subject to inherent limitations. Among conventional imaging modalities for measuring cerebral blood flow, transcranial Doppler (TCD) ultrasonography provides an indirect assessment by measuring flow velocity in major cerebral arteries. Although non-invasive, this method requires substantial operator expertise, is constrained by cranial acoustic windows, and is not well suited for prolonged continuous monitoring [5,6]. Near-infrared spectroscopy (NIRS) indirectly reflects cerebral blood flow changes by detecting local cerebral oxygenation. However, its limited penetration depth and susceptibility to scalp blood flow interference reduce its reliability for monitoring deep brain tissue hemodynamics [7]. Laser Doppler flowmetry offers a non-invasive means of assessing cerebral blood flow but is limited by relatively low temporal resolution [8]. Positron emission tomography and arterial spin labeling in magnetic resonance imaging offer high spatial and quantitative accuracy; however, their reliance on bulky, costly equipment makes them impractical for use in healthy populations or for extended, continuous monitoring [5,9,10]. In addition to imaging techniques, analytical approaches for assessing cerebral autoregulation encompass both time- and frequency-domain approaches. Time-domain quantitative indices, such as the Rate of Recovery and Autoregulation Index (ARI), provide reproducible dynamic metrics for evaluating cerebral autoregulation. However, ambiguities in parameter definitions and theoretical underpinnings, along with methodological variability across studies, limit their broader applicability [11,12]. Frequency-domain transfer function analysis quantifies cerebral autoregulation through multiple parameters, including gain, phase, and coherence; however, these parameters exhibit weak physiological interpretability [13]. Although several cerebral autoregulation assessment methods have been investigated in both animal models and clinical patients [14,15], thresholds distinguishing “normal” from “abnormal” autoregulatory states are predominantly based on prognostic outcomes. This hinders their utility in real-time, dynamic monitoring applications. Despite the current assessment methods demonstrating some consistency in specific pathological conditions, significant gaps remain in translating the findings into effective strategies for cerebral protection and clinical decision-making. This underscores the urgent need for novel technologies that offer standardization, reproducibility, and validated clinical utility.

Bioelectrical impedance technology is based on the principle that electrical impedance is a fundamental biophysical property of living tissues. In the cerebral context, electrical impedance characteristics are closely associated with the distribution state of cerebral blood flow. According to physical principles, for a cylindrical conductor of constant length, a relationship can be established between changes in electrical resistance and corresponding volume fluctuations. Consequently, impedance changes can reflect cerebral hemodynamic states during cardiac pulsation, forming the theoretical foundation of rheoencephalography (REG) [16,17,18]. This technology offers key advantages, including non-invasiveness and real-time tracking, enabling continuous monitoring of cerebral blood flow changes without the use of exogenous contrast agents or complex instrumentation. Furthermore, it provides millisecond-level temporal resolution, and its data enable the extraction of multiple parameters for in-depth assessment of cerebral autoregulation [19,20]. Tiba et al. proposed a method using trans-ocular cerebral impedance to reflect cerebral autoregulation disturbances during hypotension, thus validating the relationship between bioimpedance and blood volume [19]. However, this method was an invasive experiment conducted on large animals, and its applicability to humans requires further investigation. Further, Chen et al. found that the time difference between peaks in the blood pressure and impedance derivative signals during breath-holding intervention may reflect instantaneous changes in cerebral autoregulation [20]. While this prior study verified the effectiveness of the bioimpedance detection method for assessing cerebral autoregulation, it did not achieve quantitative assessment, nor did the authors evaluate the global regulatory efficiency of the brain. Consequently, there remains a need to develop a non-invasive method based on bioelectrical impedance technology for the quantitative assessment of global cerebral autoregulation.

To address the current absence of a “gold standard” for cerebral autoregulation assessment, this study aimed to develop a non-invasive continuous evaluation method based on bioelectrical impedance technology. Utilizing a wearable headband and Finapres device for continuous monitoring of cerebral blood flow and blood pressure, we established a graded assessment framework for cerebral autoregulation grounded in system identification principles. This framework successfully differentiated autoregulatory capacity across age groups, suggesting its potential utility for non-invasive, real-time cerebrovascular health monitoring.

## 2. Materials and Methods

### 2.1. Participants

A total of 60 healthy participants were enrolled in this study, comprising 30 young adults (aged 18–25 years) and 30 middle-aged adults (aged 50–60 years). Exclusion criteria included hypertension, diabetes mellitus, and cardiovascular, pulmonary, hepatic, or renal dysfunction, as well as habitual smoking or alcohol consumption, or the use of medications that could influence physiological measurements [14]. Hypertension was defined as a systolic blood pressure of ≥140 mm Hg or diastolic blood pressure of ≥90 mmHg. Diabetes mellitus was diagnosed based on clinical assessment or fasting blood glucose levels. Consumption of alcohol- or caffeine-containing beverages was prohibited for at least 12 h prior to experimental testing. The laboratory environment was controlled, with ambient noise minimized, temperature maintained at 24.0 ± 2.0 °C, and relative humidity at 40 ± 10%. This study was conducted in accordance with the Declaration of Helsinki and approved by the Ethics Committee of the Fourth Military Medical University (protocol code FMMU-E-III-001(2-7) and 15 January 2017).

### 2.2. Synchronized Multimodal Biosignal Acquisition

**Figure 1:**
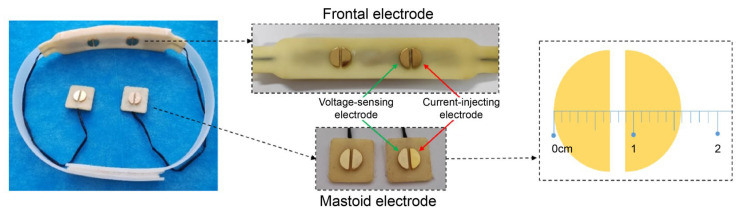
Electrode configuration and dimensional specifications of the wearable rheoencephalography (REG) monitoring headband.

Cerebral blood flow is the primary parameter required for assessing cerebral autoregulation. In this study, real-time monitoring of REG was conducted using a wearable headband, as illustrated in Figure 1. The headband comprised a main control unit, an excitation source module, an electrode module, a wireless communication module, and a power supply module. It operated at a sampling rate of 200 Hz and utilized a four-electrode configuration with constant-voltage excitation and current measurement, where the excitation voltage had a frequency of 50 kHz and an amplitude of 600 mV. The impedance acquisition module (AD5941, Analog Devices, Inc., Norwood, MA, USA) featured a signal-to-noise ratio exceeding 80 dB, with an absolute measurement error of ±0.005 Ω and a relative error of ±0.255‰.

The second essential parameter for assessing cerebral autoregulation is arterial blood pressure. In this study, blood pressure monitoring was conducted using a non-invasive continuous blood pressure monitor manufactured by Delica (Shenzhen, China), incorporating a Finapres system as the core component. This system reconstructs brachial artery systolic pressure using the Finapres vascular unloading technique, calibrated against brachial artery blood pressure recordings, thereby enabling continuous and non-invasive arterial blood pressure monitoring. It operates at a sampling frequency of 200 Hz and provides relevant hemodynamic parameters as well as indices of autonomic nervous system function for reference in research applications.

The synchronization between REG and blood pressure signals is subject to the following potential error sources and limitations: ① Hardware delays: the REG headband circuitry and Finapres device may inherently exhibit slight, yet distinct, processing delays. ② Physiological delay: an inherent physiological latency exists between systemic blood pressure changes and the subsequent impedance variations elicited in the cerebral vessels.

To mitigate the impact of these potential error sources on cerebral autoregulation assessment and to enhance the robustness of the evaluation, this study implemented a hybrid hardware–software synchronization strategy for the REG and blood pressure signals, comprising the following:

- Hardware-level synchronization: A unified clock reference was provided to both devices via Wi-Fi network time sharing.
- Software-level synchronization: Given the inherently variable nature of physiological delay, applying cross-correlation to derive a fixed delay value for correction could introduce additional errors. Therefore, timestamp-based alignment was adopted to preserve the original temporal relationship of this variable physiological delay. Based on the theoretical calculations of timestamp precision, the synchronization time difference between the two signal sets was controlled to within 2 ms.

Together with hardware synchronization, this approach ensured precise synchronization between the blood pressure and cerebral REG signals, providing a reliable data foundation for research on cerebral autoregulation.

### 2.3. Experimental Procedures

This study employed a sit-to-stand experimental paradigm to detect cerebral blood flow changes induced by alterations in cerebral perfusion pressure during rapid postural changes. First, the effectiveness of the wearable headband in capturing real-time changes in cerebral blood flow was validated. Subsequently, REG signals and non-invasive blood pressure data were further analyzed to extract parameters potentially associated with human cerebral autoregulation, enabling a functional assessment of autoregulatory performance.

**Figure 2:**
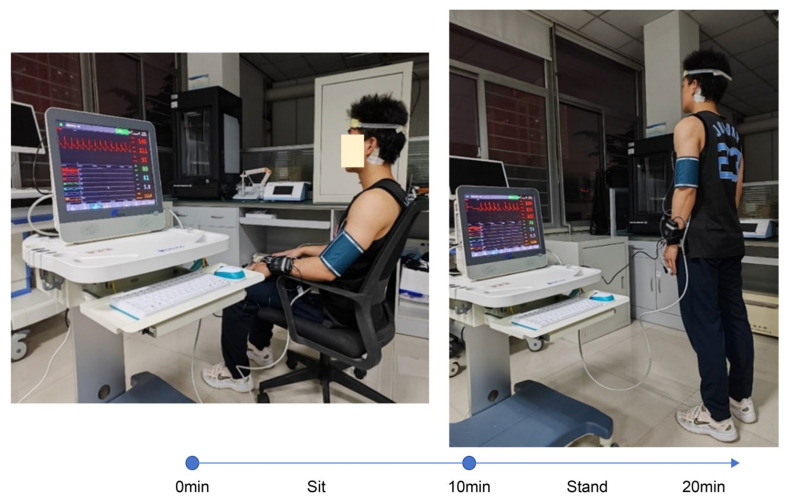
Experimental paradigm of the sit-to-stand maneuver.

First, basic participant information—such as sex, age, weight, height, and arterial blood pressure—was recorded. Subsequently, participants were guided through the experimental protocol to ensure familiarity before electrode placement. One pair of electrodes was positioned on the left forehead, 1.5 cm lateral to the cerebral midline to avoid the sagittal sinus, and at least 2 cm above the supraorbital ridge to prevent interference from the frontal sinus. The second pair of electrodes was placed on the ipsilateral mastoid process to minimize signal disruption from scalp hair. Simultaneously, the measurement module of the non-invasive blood pressure monitor was connected. Once the relevant physiological parameters stabilized, synchronized acquisition of REG and arterial blood pressure data was initiated. Following the initiation of the experiment, participants were required to undergo the sit-to-stand protocol, which involved maintaining a seated position for 10 min, followed by a rapid transition to a standing position within 2 s, and then maintaining a standing position for 10 min. This maneuver induced an instantaneous change in cerebral perfusion pressure, estimated using mean arterial pressure measured at heart level (Figure 2). Throughout the experiment, participants were instructed to keep their eyes closed, remain naturally relaxed yet awake, and maintain a normal breathing rate. Any body movements were meticulously recorded. Upon completion of the experiment, all monitoring equipment—including forehead and mastoid electrodes, as well as the finger and arm cuff modules of the non-invasive blood pressure monitor—were sequentially removed.

### 2.4. Data Pre-Processing

Dynamic assessment of cerebral autoregulation places stringent requirements on the quality of raw signals, making rigorous quality control and pre-processing of raw data essential prior to analysis. In this study, this process involved three main steps.

#### 2.4.1. Signal Quality Assessment and Artifact Processing

Before applying filtering or beat-to-beat averaging, the integrity of raw REG and non-invasive blood pressure data was reviewed. Beat-to-beat waveforms with clear morphology were retained, whereas segments containing artifacts—such as spike noise or step-like distortions caused by the blood pressure monitor self-calibration—were removed.

#### 2.4.2. Pre-Processing Stage for REG Signals

The pre-processing stage for REG signals involved several steps, including digital conversion, filtering, phase correction, and baseline drift removal to generate continuous, real-time waveforms. This was followed by feature point extraction, normalization, and additional procedures to obtain a standardized REG signal. Furthermore, to reflect cerebral blood flow changes during each cardiac cycle, the integral of the REG signal was calculated over individual cardiac cycles, as detailed below.

**Figure 3:**
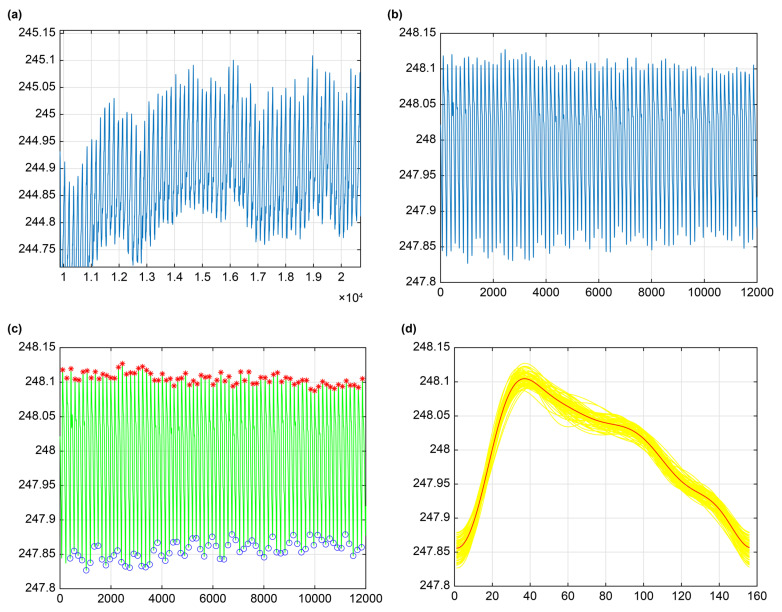
Schematic representation of REG data pre-processing (unit: Ω). (**a**) Raw cerebral impedance signal acquired during real-time monitoring; (**b**) REG waveform obtained through real-time monitoring; (**c**) Identification of characteristic points (peaks and troughs) in the REG waveform, with peaks marked by red asterisks and troughs denoted by blue circles; (**d**) Standardized REG waveform after temporal scaling and averaging, where individual REG waveforms are displayed in yellow and the resulting standardized waveform is overlaid in red.

- Reading cerebral impedance measurement data: The raw data collected by the headband were stored in hexadecimal format within a txt file. Character conversion was performed according to the Bluetooth data protocol outlined in Table 1. Each cerebral impedance data point was reconstructed to obtain the raw measurement data under real-time monitoring conditions (Figure 3a).
- Filtering cerebral impedance data. An 8th-order Butterworth low-pass filter (sampling frequency: 200 Hz; cutoff frequency: 0.5 Hz) was applied to the signal to extract the baseline impedance drift curve. Bidirectional filtering was employed to eliminate phase distortion. Subsequently, the extracted baseline impedance drift was subtracted from the original signal to obtain the pre-processed, real-time cerebral REG signal (Figure 3b).
- Standardizing REG waveforms: Characteristic cycle points within the continuous REG waveform were screened by identifying peak points (S) based on features within the rising segment. Waveform cycles that were excessively long or short were rejected using width and amplitude thresholds applied between adjacent S points. Valid waveform complexes (Tn) were then acquired using the secondarily located S points as anchors (Figure 3c). Finally, cubic spline interpolation was applied to temporally scale the waveforms. All waveform cycles were normalized to the mean cycle duration (T). These normalized REG waveforms were then superimposed and averaged to generate a standardized cerebral REG waveform (Figure 3d), in which characteristic features within a single cardiac cycle, such as the prominent primary peak and dicrotic wave, were clearly delineated.
- Calculating the cumulative effect of cerebral blood flow changes: To quantify cerebral blood flow fluctuations, the area under the curve (AUC) of the REG signal within a single cardiac cycle was calculated. Assuming the REG signal within one cycle contains N data points $Q=\left(Q_{1},\ldots Q_{i},\ldots Q_{N}\right)$, the area between each pair of adjacent data points can be approximated using the trapezoid rule. Assuming the minimum value within one cycle is denoted as minQ, and the values at two adjacent points as $Q_{i},Q_{i+1}$, the area of the corresponding trapezoid can be calculated as shown in Equations (1) and (2):

(1) $$s_{i}=\frac{\left(Q_{i}-\min Q\right)+\left(Q_{i+1}-\min Q\right)}{2}\times (i+1-i)=\frac{Q_{i}+Q_{i+1}}{2}-\min Q$$

Consequently, the total AUC for a single cardiac cycle of REG was approximated by summing the areas of all trapezoids, as given in Equations (1)–(3):

(2) $$S=\sum_{i=1}^{i=N-1}s_{i}\approx \sum_{i=1}^{i=N-1}\left(\frac{Q_{i}+Q_{i+1}}{2}-\min Q\right)$$

**Figure 4:**
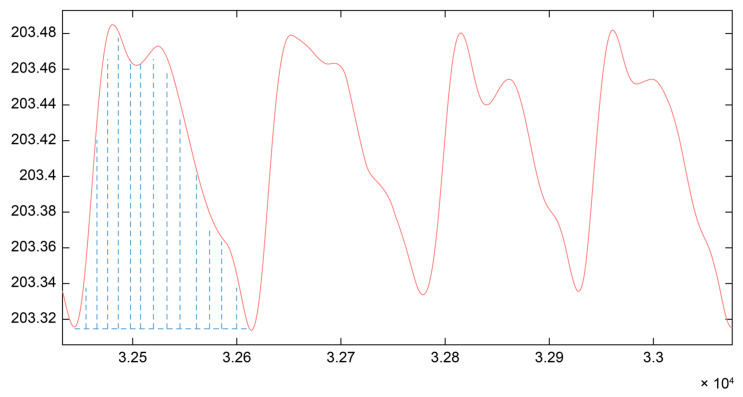
Schematic illustration of the area under the REG waveform within a single cardiac cycle (unit: Ω). The blue dashed line represents the area under the REG waveform for a single cardiac cycle.

**Figure 5:**
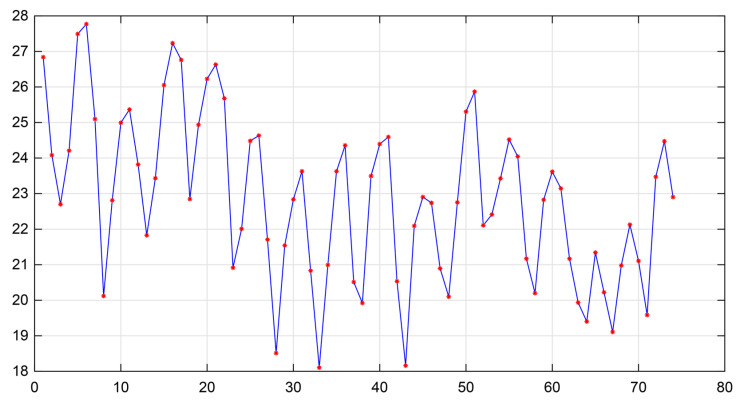
Area under the curve (AUC) of REG waveforms. Red dots indicate the AUC per waveform cycle (unit: Ω·s).

Figure 4 illustrates the area under the REG waveform for a single cardiac cycle. Using Equations (1)–(3), the AUC values of successive waveform cycles within a segment of REG data were calculated (Figure 5). This approach enabled the calculation of cerebral blood volume changes over specific time intervals.

#### 2.4.3. Beat-to-Beat Averaging of Blood Pressure Signal

Beat-to-beat averaging of the blood pressure signal was performed to reduce high-frequency oscillations caused by cardiac pulsation and emphasize the low-frequency components relevant to cerebral autoregulation.

### 2.5. Linear Time-Invariant Analytical Model

Cerebral autoregulation was modeled as a linear time-invariant (LTI) system, and time-domain analytical methods were employed using corresponding algorithms in conjunction with experimental data.

Physiologically, cerebral autoregulation primarily stabilizes cerebral blood flow by dynamically adjusting cerebrovascular resistance. When blood pressure changes, cerebral blood vessels adjust their resistance through vasoconstriction or vasodilation to maintain cerebral blood flow near a target level. According to Poiseuille’s hydrodynamic law, cerebral blood flow *Q*(*t*) can be expressed as Equation (3):

(3) $$Q\left(t\right)=\frac{P\left(t\right)-P_{venous}}{R\left(t\right)}\approx \frac{P\left(t\right)}{R\left(t\right)}$$

where *P*(*t*) represents the mean arterial pressure and *R*(*t*) denotes the cerebrovascular resistance. The venous pressure *P*_venous_ is typically low and thus can be neglected. Given that cerebral autoregulation functions to maintain cerebral blood flow *Q*(*t*) near a target value *Q*_0_ by adjusting cerebrovascular resistance *R*(*t*), the rate of change of resistance *R*(*t*) is proportional to the deviation of cerebral blood flow *Q*(*t*) from *Q*_0_. Consequently, the dynamics of resistance change can be described by Equation (4):

(4) $$\frac{dR}{dt}=k\left(\frac{P\left(t\right)}{R\left(t\right)}-Q_{0}\right)$$

where *k* denotes the regulatory rate constant. According to the principle of negative feedback control, when *Q*(*t*) > *Q*_0_, the rate of change d*R*/d*t* > 0, indicating an increase in vascular resistance; conversely, when *Q*(*t*) < *Q*_0_, d*R*/d*t* < 0, indicating a decrease in vascular resistance.

Given that the resistance *R*(*t*) appears in the denominator of Equation (4), the resulting expressions include non-linear terms, rendering the model inherently non-linear. To address this, the non-linear model was first linearized, thereby enabling its transformation into a linear system. The linearization method typically involved performing a Taylor series expansion around a steady-state operating point and omitting higher-order small terms, thereby yielding an approximate linear model. Assuming the system operates near a steady-state point, the key variables can be expressed as *P*(*t*) = *P*_0_ + Δ*P*(*t*), *R*(*t*) = *R*_0_ + Δ*R*(*t*), and *Q*(*t*) = *Q*_0_ + Δ*Q*(*t*), where *P*_0_, *R*_0_, and *Q*_0_ denote steady-state values, and Δ*P*, Δ*R*, and Δ*Q* represent small perturbations relative to the steady state. Substituting *R*(*t*) = *R*_0_ + Δ*R*(*t*) and *P*(*t*) = *P*_0_ + Δ*P*(*t*) into Equation (3) and applying a Taylor series expansion while neglecting higher-order small terms (such as Δ*R*^2^ and Δ*P*Δ*R*), we derived the expressions in Equations (5) and (6):

(5) $$\frac{P\left(t\right)}{R\left(t\right)}=\frac{P_{0}+\Delta P\left(t\right)}{R_{0}+\Delta R\left(t\right)}\approx \frac{P_{0}}{R_{0}}\left(1+\frac{\Delta P\left(t\right)}{P_{0}}\right)\left(1-\frac{\Delta R\left(t\right)}{R_{0}}\right)$$

(6) $$\frac{P\left(t\right)}{R\left(t\right)}\approx \frac{P_{0}}{R_{0}}+\frac{\Delta P\left(t\right)}{R_{0}}-\frac{P_{0}\Delta R\left(t\right)}{R_{0}^{2}}$$

Given that *Q*_0_ = *P*_0_/*R*_0_, the original resistance dynamics in Equation (4) can be simplified to Equation (7):

(7) $$\frac{d\left(R_{0}+\Delta R\right)}{dt}=k\left(\left(Q_{0}+\frac{\Delta P}{R_{0}}-\frac{P_{0}\Delta R}{R_{0}^{2}}\right)-Q_{0}\right)$$

Further simplification yielded Equation (8):

(8) $$\frac{d\Delta R}{dt}=k\left(\frac{\Delta P}{R_{0}}-\frac{P_{0}\Delta R}{R_{0}^{2}}\right)$$

At this point, the change in resistance Δ*R* can be expressed as a function of cerebral blood flow change Δ*Q*. Applying this to the linearized cerebral blood flow equation (Equation (6)) and again neglecting higher-order small terms resulted in Equation (9)

(9) $$Q\left(t\right)=\frac{P\left(t\right)}{R\left(t\right)}\approx Q_{0}+\Delta Q=\frac{P_{0}+\Delta P}{R_{0}+\Delta R}\approx Q_{0}+\frac{\Delta P}{R_{0}}-\frac{Q_{0}\Delta R}{R_{0}}$$

Simplification yielded Equation (10):

(10) $$\Delta Q\approx \frac{\Delta P}{R_{0}}-\frac{Q_{0}}{R_{0}}\Delta R$$

Solving for Δ*R* resulted in Equation (11):

(11) $$\Delta R=\frac{\Delta P}{Q_{0}}-\frac{R_{0}}{Q_{0}}\Delta Q$$

Substituting Δ*R* into the resistance dynamics (Equation (8)) yielded Equation (12):

(12) $$\frac{d}{dt}\left(\frac{\Delta P}{Q_{0}}-\frac{R_{0}}{Q_{0}}\Delta Q\right)=\frac{k}{R_{0}}\Delta P-\frac{kQ_{0}}{R_{0}}\left(\frac{\Delta P}{Q_{0}}-\frac{R_{0}}{Q_{0}}\Delta Q\right)$$

The time derivative on the left-hand side corresponds to Equation (13):

(13) $$\frac{d}{dt}\left(\frac{\Delta P}{Q_{0}}\right)-\frac{R_{0}}{Q_{0}}\frac{d\Delta Q}{dt}=\frac{1}{Q_{0}}\frac{d\Delta P}{dt}-\frac{R_{0}}{Q_{0}}\frac{d\Delta Q}{dt}$$

The expression on the right-hand side is given by Equation (14):

(14) $$\frac{k}{R_{0}}\Delta P-k\frac{Q_{0}}{R_{0}}\frac{\Delta P}{Q_{0}}+k\frac{Q_{0}}{R_{0}}\cdot \frac{R_{0}}{Q_{0}}\Delta Q=\frac{k}{R_{0}}\Delta P-\frac{k}{R_{0}}\Delta P+k\Delta Q=k\Delta Q$$

Substituting these into the previous formulation yielded the governing equation shown in Equation (15):

(15) $$\frac{1}{Q_{0}}\frac{d\Delta P}{dt}-\frac{R_{0}}{Q_{0}}\frac{d\Delta Q}{dt}=k\Delta Q$$

Rearranging the terms resulted in Equation (16):

(16) $$\frac{d\Delta Q}{dt}+\frac{kQ_{0}}{R_{0}}\Delta Q=\frac{1}{R_{0}}\frac{d\Delta P}{dt}$$

This represents a first-order linear ordinary differential equation, which can be expressed as Equation (17):

(17) $$\tau \frac{d\Delta Q}{dt}+\Delta Q=G\frac{d\Delta P}{dt}$$

Here, the time constant (*τ*) and system gain (*G*) are defined as Equations (18) and (19):

(18) $$\tau =\frac{R_{0}}{kQ_{o}}$$

(19) $$G=\frac{1}{kQ_{0}}$$

The time constant *τ* characterizes the responsiveness of the regulatory mechanism—that is, the speed at which it reacts to changes in blood pressure. For example, when blood pressure rises abruptly, cerebral vessels require a certain amount of time to constrict and stabilize blood flow. A smaller time constant *τ* indicates a faster vasoconstrictive response, enabling the system to return to a steady state more rapidly. Conversely, a larger *τ* signifies a slower response, potentially resulting in prolonged fluctuations in cerebral blood flow during pressure changes, which may increase the risk of cerebral injury.

The gain *G* reflects the sensitivity of the system and is typically defined as the ratio of output to input. In this context, it quantifies the extent of cerebral blood flow change induced by a unit change in blood pressure. A gain *G* value near zero indicates intact regulatory capacity, where cerebral blood flow remains stable despite changes in blood pressure. However, a higher gain indicates weaker regulatory capacity, in which changes in blood pressure lead to significant variations in cerebral blood flow. Therefore, smaller values of the time constant *τ* and gain *G* indicate a more rapid regulatory response and more effective steady-state control of cerebral blood flow. Conversely, larger values suggest a slower response or steady-state dysregulation. These results suggest that both time constant *τ* and gain *G* can serve as theoretical indicators of cerebral autoregulation performance, with Equation (17) offering an LTI system model for their theoretical characterization.

This study assessed cerebral autoregulation from a time-domain perspective, primarily applying linear analytical methodologies. Although the relationship between arterial blood pressure and cerebral blood volume is inherently non-linear, linear methods can effectively capture the core dynamics of cerebral autoregulation under appropriate assumptions [21]. Based on this LTI model, a corresponding computational algorithm was implemented using MATLAB R2021b.

## 3. Results

**Figure 6:**
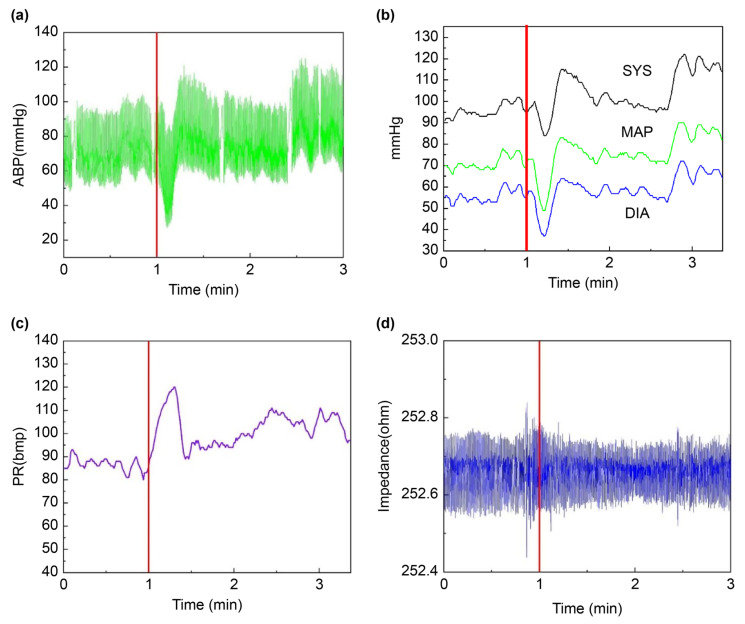
Trends in key physiological parameters during the sit-to-stand maneuver. (The red vertical line indicates the moment of standing up, with coordinates of 1 min). (**a**) Arterial blood pressure is shown in green; (**b**) Systolic, diastolic, and mean arterial pressures are displayed in black, blue, and green, respectively; (**c**) Heart rate is depicted in purple; (**d**) REG waveform is presented in blue.

During the sit-to-stand maneuver paradigm in this study, postural changes induced a transient decrease in arterial blood pressure. Notably, systolic and diastolic blood pressures, as well as mean arterial pressure, all demonstrated this trend. Simultaneously, heart rate showed a significant increase, as shown in Figure 6.

A comparison of the data between the seated (0~10 min) and sit-to-stand transition (10~20 min) periods revealed no statistically significant change in the amplitude of cerebral impedance measurements. In contrast, a significant increase was observed in mean arterial pressure, systolic and diastolic blood pressures, and heart rate (*p* < 0.05) (Table 2). After a sudden change in the participant’s body position, blood was retained in the lower limbs and abdomen owing to gravity, resulting in a decrease in venous return flow. Healthy people compensate through the stress reflex, including an increase in heart rate to maintain cardiac output, and peripheral vasoconstriction leading to a rise in diastolic and mean arterial pressure [5,22].

Although no significant changes were observed in the cerebral impedance data throughout the 20-min experiment, the impedance waveform was not static during the standing period. From a temporal analysis perspective, determining the time required for cerebral blood flow to return to a steady state—specifically, the speed at which it stabilizes following a change in cerebral perfusion pressure—is of significant interest. To this end, this study proposed a novel analysis method based on the REG recovery curve. This method employed time-domain correlation operations to obtain the REG recovery curve, enabling precise estimation of the time ‘t’ required for the REG signal to transition from one equilibrium state to another.

**Figure 7:**
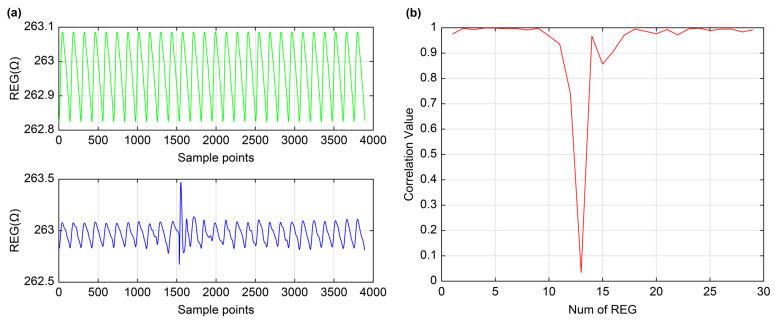
Extraction of temporal parameters from recovery curves. (**a**) Top: standardized REG waveform (green), Bottom: REG waveform alterations during the sit-to-stand maneuver paradigm (blue); (**b**) Recovery curve (red) showing pronounced fluctuations during the sit-to-stand paradigm.

The recovery curve was generated by performing a time-domain correlation between the real-time REG signal recorded during the task paradigm and the standardized REG signal. In this curve, the abscissa represented time, whereas the ordinate represented the correlation value. A correlation peak value of 1 indicated that the REG signal had fully returned to its pre-task equilibrium state. The interval between the onset of the task and the point at which the recovery curve was stabilized reflected the time required for cerebral blood flow to return to a steady state. A shorter time indicated faster cerebral blood flow recovery, whereas a longer time indicated slower recovery. As illustrated in Figure 7, the sit-to-stand maneuver induced marked amplitude fluctuations and significant morphological changes in the REG waveform, which were clearly reflected in the corresponding variations of the recovery curve.

Statistical analysis revealed that cerebral blood flow returned to normal after 9 ± 0.82 waveform cycles in the healthy young group and 11.67 ± 1.15 waveform cycles in the healthy middle-aged group, with a statistically significant difference in recovery time between the two groups. Therefore, the characteristics of the recovery curve can serve as an indicator for assessing cerebral blood flow recovery capacity across different populations in response to blood pressure changes.

**Figure 8:**
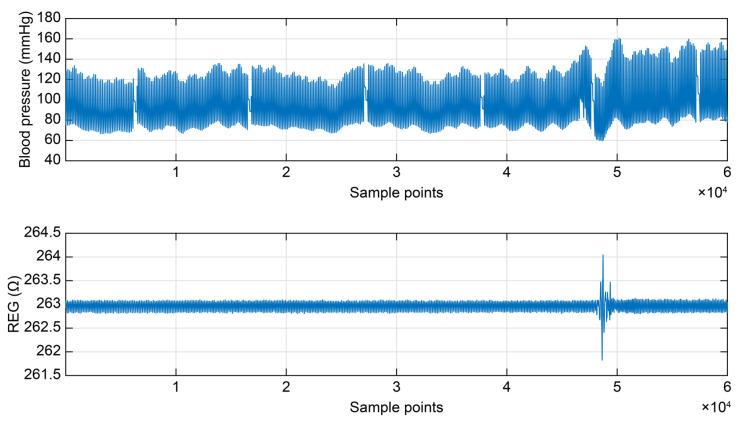
Simultaneous acquisition of blood pressure and REG data.

**Figure 9:**
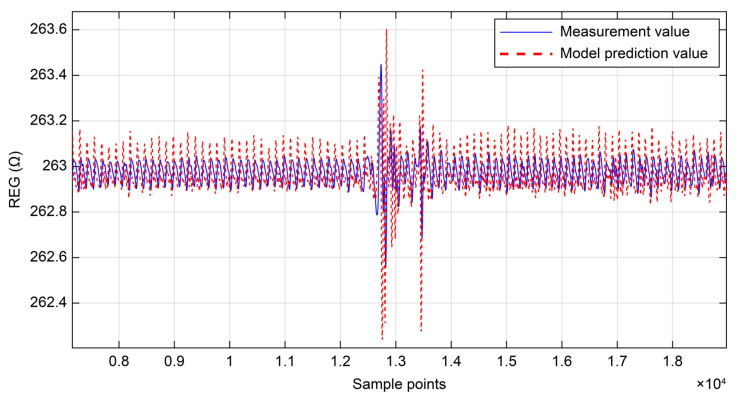
Comparison of model-predicted and experimentally measured cerebral blood flow (partial view: red line = predicted values, blue = measured values; the sampling frequency of the headband is 200 Hz, so the time interval for one sampling point is 5 ms).

To further quantify cerebral autoregulation, a first-order linear differential equation was employed from a systems perspective to describe the dynamic response of the system. For this aspect of this study, blood pressure and REG data were acquired from participants (Figure 8). Segments of 300 s were selected from both signals, each sample at 200 Hz, yielding a total of 60,000 data points per signal. Based on the solution of the LTI model, a comparison was made between the cerebral blood flow values measured by the headband via REG and those predicted by the cerebral autoregulation model. The model-predicted values of cerebral blood flow closely matched the actual measured values (Figure 9). The root mean square error (RMSE) and the coefficient of determination (R^2^) were used as evaluation metrics to assess the predictive performance of the linear regression model. The analysis revealed a RMSE value of <0.1, indicating a small average error between the model predictions and the actual values. Simultaneously, R^2^ was >0.9, approaching 1, which indicated a good fit of the model to the data, explaining most of the variance in the dependent variable.

**Figure 10:**
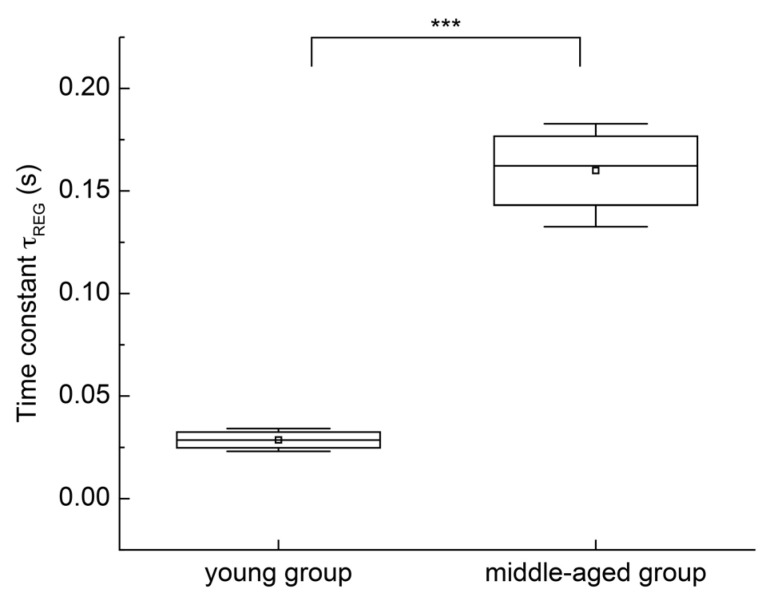
Statistical analysis results of time constants between young and middle-aged groups (*n* = 30). (***: *p* < 0.001).

The Shapiro–Wilk test was applied to the time constant samples of both groups. *p*-values greater than 0.05 were obtained for the young group (*p* = 0.1185) and middle-aged group (*p* = 0.1069). Therefore, the normality assumption was accepted for both datasets, and an independent sample *t*-test was performed. The specific analysis results are shown in Figure 10. The time constants (*τ*_REG_) for the young and middle-aged groups were 0.0696 ± 0.005 s and 0.2006 ± 0.0045 s, respectively. Statistical analysis indicated a significant difference between the two groups (*p* < 0.001). The time constant *τ*_REG_ increased progressively with age, indicating a significant age-related effect. Furthermore, the Shapiro–Wilk test was applied to the gain (*G*_REG_) samples of both groups. *p*-values greater than 0.05 were obtained for the young group (*p* = 0.1322) and the middle-aged group (*p* = 0.1441). Therefore, the normality assumption was accepted for both datasets, and an independent sample *t*-test was performed. The gains (*G*_REG_) for the young and middle-aged groups were (2.7355 × 10^−9^) ± (2.5114 × 10^−9^) Ω/mmHg and (0.001) ± (8.0986 × 10^−5^) Ω/mmHg, respectively. Statistical analysis indicated a significant difference between the two groups (*p* < 0.001). The gain G_REG_ increased progressively with age, indicating a significant age-related effect.

## 4. Discussion

The findings of this study demonstrate that cerebral autoregulation exhibits measurable differences across age groups, as evidenced by both temporal and systems-level analyses. From a temporal perspective, the cerebral blood flow recovery time was shorter in the young group than in the middle-aged group, indicating a stronger regulatory capacity in younger individuals in response to blood pressure fluctuations. From the systems perspective, time-domain analysis using an LTI model employed the time constant *τ*_REG_ to quantify the dynamic response speed of the autoregulation system, reflecting the strength of cerebral autoregulation capability. The results revealed a significant difference in *τ*_REG_ between the young and middle-aged groups. The young group exhibited a shorter time constant, *τ*_REG_, indicating a faster regulatory speed, quicker compensation for blood pressure fluctuations, and a stronger capacity to maintain stable cerebral blood flow. In contrast, a prolonged *τ*_REG_ may reflect impaired regulatory function, predisposing individuals to transient cerebral blood flow fluctuations, as observed among older adults or patients with cerebrovascular diseases [1]. These results align with recent findings by Panerai, who reported that ARI—a parameter used to assess cerebral autoregulation capability—declines with age, specifically decreasing by 0.025 units per year in males (*p* = 0.022) [23]. Overall, these findings suggest that the time constant *τ*_REG_ can serve as a quantitative marker for distinguishing cerebral autoregulatory function between the young and middle-aged groups, enabling not only qualitative analyses but also potentially facilitating quantitative assessments.

Although an age-related cerebral autoregulation decline is well-established [23,24,25], this study establishes a novel quantitative index based on wearable REG technology, *τ*_REG_, aimed at quantifying this physiological process in a non-invasive, continuous, and dynamic manner. Our findings demonstrate a significantly larger *τ*_REG_ in middle-aged adults, which aligns with established knowledge of age-related vascular changes including: (1) reduced vascular elasticity and increased stiffness [25]; (2) imbalance in vasoactive substances (e.g., decreased bioavailability of nitric oxide, which mediates vasodilation) [26]; (3) impaired metabolic regulation efficiency (e.g., reduced CO_2_ reactivity) [2]; and (4) a decline in autonomic nervous system regulatory function [24]. We hypothesize that the observed increase in *τ*_REG_ would occur as a direct consequence of these mechanistic changes: increased stiffness reduces compliance, while endothelial and metabolic dysfunction diminish the efficacy and speed of the vasomotor response, collectively resulting in a prolonged system time constant.

Although the current study establishes the association between *τ*_REG_ and age, the underlying biological mediators warrant further investigation. Consequently, a key priority for future work will involve focusing on investigating the correlation between *τ*_REG_ and gold-standard indicators (such as vascular stiffness (PWV) [27], endothelial function (FMD) [28], and metabolic regulation (TCD-CO_2_ test) [29]) to further elucidate the physiological significance of *τ*_REG_ and to consolidate its clinical applicability. To further establish the clinical validity and reliability of the *τ*_REG_ metric, our future research agenda includes conducting direct comparative studies to evaluate its agreement and diagnostic concordance with established parameters, including the TCD-based ARI and NIRS-derived parameters.

## 5. Conclusions

In this study, we conducted exploratory research on the assessment of cerebral autoregulation using bioelectrical impedance technology. Both functional and structural parameters of cerebral autoregulation were evaluated, successfully achieving stratification between middle-aged and young cohorts within the healthy population. Moreover, the results of this study may serve as a foundation for future applications in cerebrovascular health monitoring and early risk warning based on cranial electrical impedance technology.

The distinctive features and innovations of this study included the development of a portable, non-invasive wearable detection system for assessing cerebral autoregulation, which enabled synchronized monitoring and pre-processing of cranial electrical impedance and arterial blood pressure signals. By integrating measurement data from REG and arterial blood pressure, we established a time-domain analytical method based on an LTI system. Notably, this study is the first to utilize a wearable REG device within a dynamic sit-to-stand paradigm to quantify age-related differences in cerebral autoregulation in healthy adults. Compared to the mainstream time-domain ARI model [12], the time constant (*τ*_REG_) serves as a quantitative indicator with clear physical significance, directly related to the response speed of the system. This provides a potential tool for assessing vascular regulatory function in more naturalistic states.

To advance cerebrovascular health monitoring and early risk warning based on cranial electrical impedance technology, further investigations are required. These include applying the established cerebral autoregulation analysis and assessment methods to participant cohorts with broader demographic coverage to enhance the reliability and validity of the findings. Specifically, future efforts should focus on the following:

- Further optimization of the assessment of cerebral blood flow regulation based on bioelectrical impedance technology could be achieved by introducing advanced algorithms, such as deep learning models [30], and by exploring the integration of deep learning techniques to improve signal quality during dynamic periods and enhance the utilization of limited datasets;
- Promote the application of the analytical method, with a focused effort on clinical populations with cerebrovascular diseases, to enable long-term monitoring and early screening in high-risk groups (e.g., patients with stroke, vascular dementia);
- Develop a more miniaturized and comfortable wearable multimodal system (headband + Finapres) to extend its functionality toward continuous dynamic monitoring beyond laboratory settings.

Subsequent improvements to the assessment model and methodology should be guided by the outcomes of these extended experimental validations.

## Figures and Tables

**Table 1:** Definition of Bluetooth data packet structure.

Name	Content	Remarks
Start character	68H	Start identifier
Packet length	XX XX	2-byte short integer
Function code	27H	Left hemisphere initiation
Data field	4 × CNT bytes	Payload data
CRC checksum	XX XX	2-byte checksum
End character	16H	End identifier

**Table 2:** Comparison of key physiological parameters between seated and sit-to-stand states.

Protocol	Seated	Sit-to-Stand Maneuver	*p* Value
Left brain bioimpedance (ohm)	212.2 ± 33.03	210.1 ± 28.3	0.77
Mean arterial pressure (mmHg)	76.1 ± 13.0	89.2 ± 8.9	<0.05
Systolic blood pressure (mmHg)	108.7 ± 18.5	121.0 ± 15.0	<0.05
Diastolic blood pressure (mmHg)	52.9 ± 9.0	67.0 ± 8.3	<0.05
Heart rate (beats/min)	75.2 ± 8.7	84.6 ± 13.6	<0.05

## Data Availability

The raw data supporting the conclusions of this article will be made available by the authors on request.

## Abbreviations

The following abbreviations are used in this manuscript:

REG	Rheoencephalography
*τ*_REG_	Time Constant of Cerebral Autoregulation
ARI	Autoregulation Index
LTI	Linear Time-Invariant
RMSE	Root Mean Square Error
R^2^	Coefficient of Determination (R-squared)
